# Theoretical Study of the Thermolysis Reaction and Chemiexcitation of Coelenterazine Dioxetanes

**DOI:** 10.1021/acs.jpca.2c01835

**Published:** 2022-05-25

**Authors:** Carla M. Magalhães, Joaquim C. G. Esteves da Silva, Luís Pinto da Silva

**Affiliations:** † Chemistry Research Unit (CIQUP), Institute of Molecular Sciences (IMS), Faculty of Sciences of University of Porto (FCUP), Rua do Campo Alegre 687, 4169-007 Porto, Portugal; ‡ LACOMEPHI, GreenUPorto, Department of Geosciences, Environment and Territorial Planning, Faculty of Sciences of University of Porto (FCUP), Rua do Campo Alegre 687, 4169-007 Porto, Portugal

## Abstract

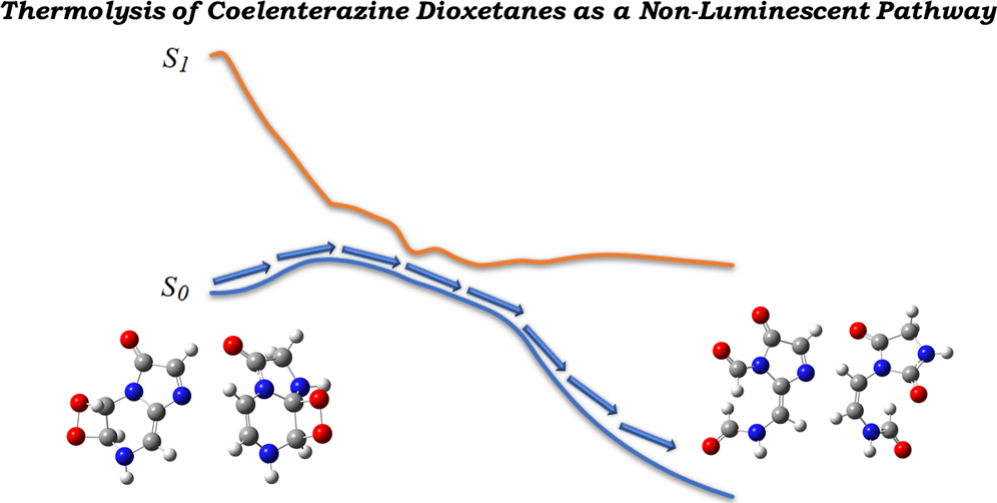

Coelenterazine and other imidazopyrazinones are important bioluminescent substrates widespread in marine species and can be found in eight phyla of luminescent organisms. Light emission from these systems is caused by the formation and subsequent thermolysis of a dioxetanone intermediate, whose decomposition allows for efficient chemiexcitation to singlet excited states. Interestingly, some studies have also reported the involvement of unexpected dioxetane intermediates in the chemi- and bioluminescent reactions of Coelenterazine, albeit with little information on the underlying mechanisms of these new species. Herein, we have employed a theoretical approach based on density functional theory to study for the first time the thermolysis reaction and chemiexcitation profile of two Coelenterazine dioxetanes. We have found that the thermolysis reactions of these species are feasible but with relevant energetic differences. More importantly, we found that the singlet chemiexcitation profiles of these dioxetanes are significantly less efficient than the corresponding dioxetanones. Furthermore, we identified triplet chemiexcitation pathways for the Coelenterazine dioxetanes. Given this, the chemiexcitation of these dioxetanes should lead only to minimal luminescence. Thus, our theoretical investigation of these systems indicates that the thermolysis of these dioxetanes should only provide “dark” pathways for the formation of nonluminescent degradation products of the chemi- and bioluminescent reactions of Coelenterazine and other imidazopyrazinones.

## Introduction

**Scheme 1:**
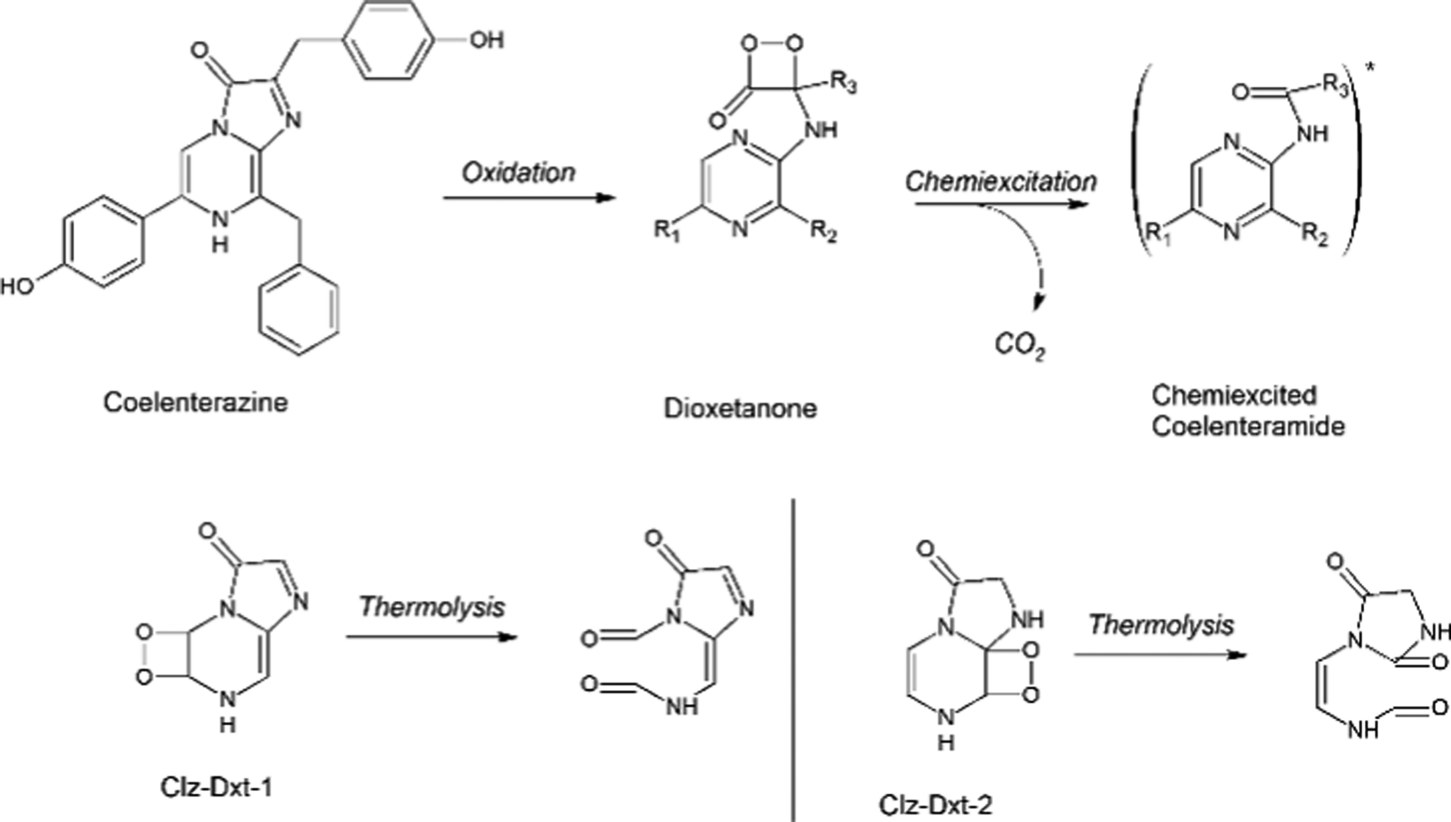
Schematic Depiction of the Chemi- and Bioluminescent Reactions of Coelenterazine (Top) and Schematic Depiction of the Thermolysis of Clz-Dxt-1 and Clz-Dxt-2 (Bottom)^17,21^

Bioluminescence is a remarkable phenomenon in which light is emitted due to a biochemical reaction in living organisms.^1,2^ Bioluminescence is widespread in nature, with more than 700 genera generating this type of light emission.^3−5^ In fact, bioluminescence can be observed in organisms as distinct as fireflies, fungi, and earthworms.^3−5^ Interestingly, ∼80% of luminescent organisms can be found in the oceans,^6,7^ with many of them utilizing the same class of compounds as bioluminescent substrates: imidazopyrazinones.^8−10^ Among them, Coelenterazine (Scheme 1) is arguably the most widespread bioluminescent imidazopyrazinone in marine species.^6−10^

Typical bioluminescent reactions involving Coelenterazine proceed via the following general mechanism (Scheme 1): first, the oxygenation of the imidazopyrazinone core of Coelenterazine occurs, with the subsequent formation of a cyclic peroxide termed dioxetanone;^11^ the latter high-energy intermediate is essential for chemiexcitation as its thermolysis allows for a thermally activated singlet ground state (*S*_0_) to produce an oxidized reaction product (Coelenteramide) in its first singlet excited state (*S*_1_).^9−13^*S*_1_ Coelenteramide is then responsible for light emission by radiative decay to its *S*_0_ state.^14,15^

While the light emission process that occurs during Coelenterazine-based bioluminescence is relatively well characterized, the pathways that lead to Coelenterazine degradation products via “dark” reactions are still far from being understood. For example, Inouye et al. studied the degradation process of (*S*)-2-peroxycoelenterazine in aequorin under conditions of protein denaturation and found that by acid treatment the major product was Coelenteramine and not Coelenteramide (with no significant luminescence).^16^ Burakova et al. also found recently some unexpected Coelenterazine degradation products of *Beroe abyssicola* photoprotein photoinactivation.^17^ One of these degradation products was identified as a substituted hydantoin compound, termed 4*Z*/*E* (Scheme 1).^17^ Interestingly, Burakova et al. attributed the formation of 4*Z*/*E* for the formation and subsequent thermolysis of a cyclic peroxide intermediate, but for which identity was not of the most common Coelenterazine dioxetanone species rather that of an unexpected Coelenterazine dioxetane compound.^17^ This report is of note as while dioxetanes are similar to dioxetanones, in which both high-energy intermediates are capable of chemiexcitation,^18−20^ the former ones have not been associated with the bioluminescent reactions of Coelenterazine. Furthermore, while dioxetanes are capable of efficient singlet chemiexcitation,^18,19^ the study of Burakova et al. did not report the thermolysis of Coelenterazine dioxetane as a luminescent pathway.^17^

It should be noted that while rare, the study of Burakova et al. was not the first to mention a Coelenterazine dioxetane species and its respective thermolysis.^17^ In 1996, Teranishi et al. studied the chemiluminescent properties of a hydroperoxide derivative of Coelenterazine (Scheme 1) and found that it decomposed via the formation of a dioxetane intermediate (a “mirror” species of that proposed by Burakova et al.) but without relevant luminescence.^21^ Thus, these reports indicate that besides Coelenterazine dioxetanone, its dioxetane counterpart can also play a role in the bioluminescent and chemiluminescent reactions of Coelenterazine. Furthermore, while Coelenterazine dioxetanone is responsible for chemiexcitation and subsequent light emission, the Coelenterazine dioxetanes appear to lead only to degradation products with minimal luminescence.

However, there are still significant questions regarding Coelenterazine dioxetanes. Namely, what is the reaction mechanism of their thermolysis reactions, and, more importantly, why these dioxetanes appear to be unable to efficiently generate luminescence (contrary to Coelenterazine dioxetanone)? To address these questions, we will study for the first time the thermolysis and potential chemiexcitation of Coelenterazine dioxetane species. To this end, we will employ a reliable and accurate theoretical approach based on time-dependent (TD) density functional theory (DFT).^9,10,12,22,23^ With this study, we intend to evaluate the role of dioxetane in the bioluminescent and chemiluminescent reactions of Coelenterazine: information that could be expanded to other systems as the imidazopyrazinone core of this substrate can be found in eight phyla of bioluminescent organisms.

## Computational Details

DFT and TD-DFT calculations were performed with long-range-corrected hybrid exchange–correlation functional ωB97XD.^24^ This functional, as well as other long-range-corrected hybrid exchange–correlation density functionals, has been used with success in the study of different dioxetanone and dioxetane systems.^9−12,22,23,25,26^ Furthermore, ωB97XD has also been shown to provide reliable estimates for π → π*, *n* → π*, and local excitations and charge-transfer (CT) and Rydberg states.^27^ Geometries and frequency calculations of the *S*_0_ state of Coelenterazine dioxetane species were obtained at the ωB97XD/6-31G(d,p) level of theory, with an open-shell (U) approach for transition states (TSs) and a closed-shell (R) one for reactants/products. The broken-symmetry technique was also used with the U approach for generating an initial guess for a biradical.^9−12,22,23,25,26^ IRC calculations were performed at the same level of theory to determine whether the obtained TSs connected with the desired reactants and products. The Cartesian coordinates of the obtained TSs are presented in Tables S1 and S2. The *S*_0_ energies of the IRC-obtained structures were evaluated by single-point calculations at the U ωB97XD/6-31+G(d,p) level of theory. The energies of the corresponding *S*_1_ state were calculated by TD ωB97XD/6-31+G(d,p) calculations on top of the *S*_0_ structures. Finally, the energies of the corresponding first triplet excited states (*T*_1_) were also obtained by U ωB97XD/6-31+G(d,p) calculations on top of the *S*_0_ IRC-obtained structures. These calculations were performed in the gas phase to understand the intrinsic properties of these systems without being perturbed by the external microenvironment. Calculations were performed with the Gaussian 09 software package.^28^ The employed theoretical protocol was already used with success in the study of this type of system.^9−12,22,23,25,26,29,30^

## Results and Discussion

In this study, we intend to elucidate the chemical details behind the thermolysis and potential chemiexcitation of the two Coelenterazine dioxetane species proposed by both Teranishi et al.^21^ and Burakova et al.,^17^ as found in Scheme 1. From now on, these dioxetane species will be termed Clz-Dxt-1 and Clz-Dxt-2, respectively. To achieve a compromise between accuracy and computational cost, we focused our study on the imidazopyrazinone scaffold of these species by substituting the large and more flexible substituents of Coelenterazine (depicted as R_1_, R_2_, and R_3_ in Scheme 1) with hydrogen atoms. This type of substitution is expected to retain the intrinsic characteristics of these systems, as found previously for imidazopyrazinone-based systems.^10,22,25,29−33^ We have also focused on the neutral forms of these species, as Burakova et al. attributed this decomposition pathway to neutral Clz-Dxt-2.^17^ Furthermore, Teranishi et al. found that Clz-Dxt-1 leads to higher luminescence in acidic media, pointing out to the involvement of a neutral species.^21^ Finally, Nery et al. have previously indicated that neutral dioxetanes are linked with low luminescence,^35,36^ which is compatible with the thermolysis of Clz-Dxt-1 and Clz-Dxt-2 being involved mainly with “dark” reactions.

**Figure 1:**
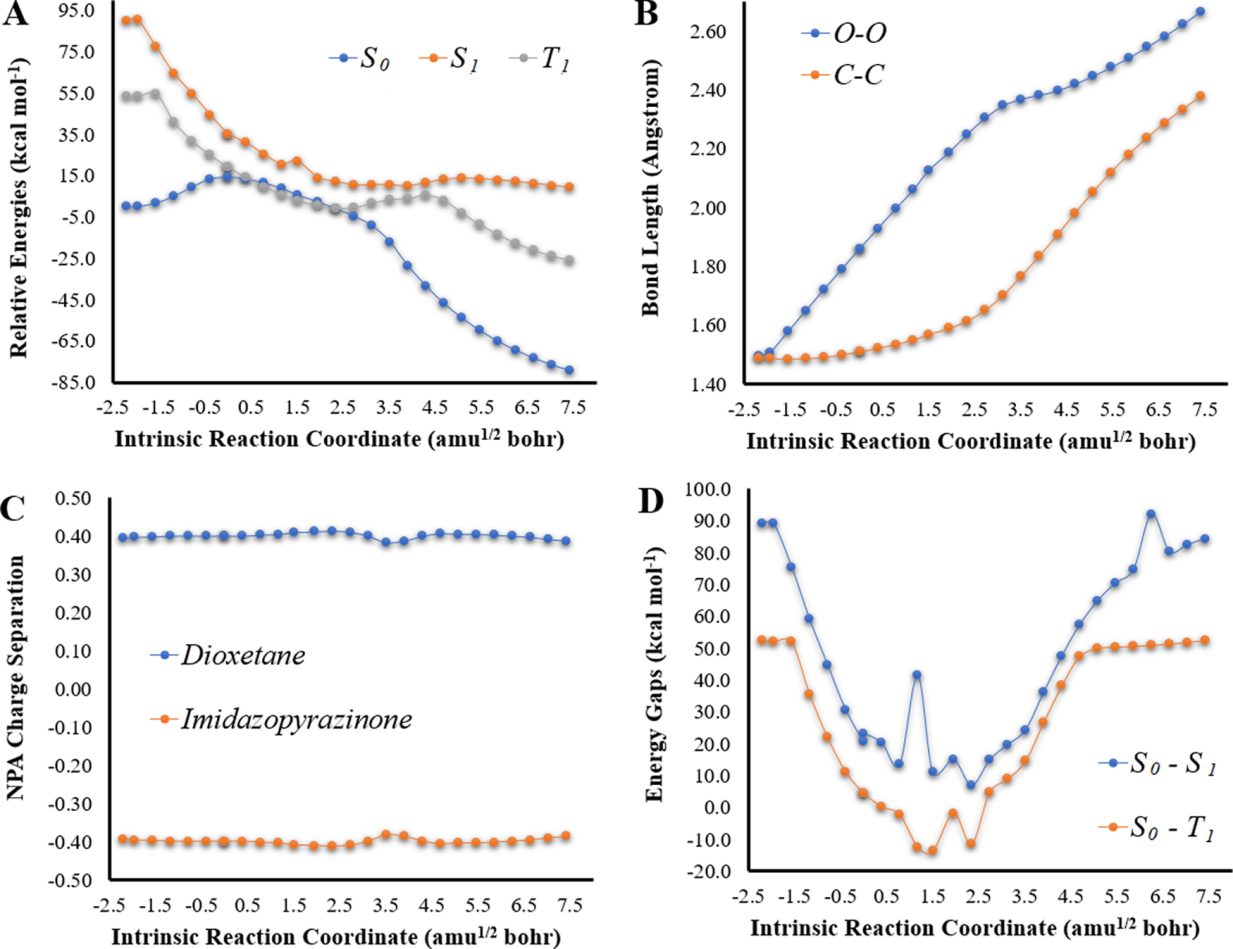
(A) Potential energy curves for the *S*_0_, *S*_1_, and *T*_1_ states of Clz-Dxt-1 in the gas phase, as a function of intrinsic reaction coordinates, at the (TD) ωB97XD/6-31+G(d,p) level of theory. (B) Bond length variation for O–O and C–C bonds of the dioxetane ring for Clz-Dxt-1, as a function of intrinsic reaction coordinates, at the ωB97XD/6-31G(d,p) level of theory. (C) NPA *S*_0_ charge separation between the dioxetane and imidazopyrazinone moieties of Clz-Dxt-1, as a function of intrinsic reaction coordinates, at the ωB97XD/6-31+G(d,p) level of theory. (D) *S*_0_–*S*_1_ and *S*_0_–*T*_1_ energy gaps (in kcal mol^–1^) for Clz-Dxt-1 in implicit diethyl ether, as a function of intrinsic reaction coordinates, at the (TD) ωB97XD/6-31+G(d,p) level of theory.

The first step of this study was then to evaluate the *S*_0_ thermolysis reaction of Clz-Dxt-1, whose associated potential energy curves and variations of relevant geometrical parameters are shown in Figure 1. The activation energy (Δ*E*_act_) and the energy difference between the product and reactant (Δ*E*_P–R_) are given in Table 1. The product structure used to obtain this parameter was in the *E*-conformation, as it is the direct product of the thermolysis reaction.

**Table 1:** Activation Energies (Δ*E*_act_, in kcal mol^–1^) and Energy Differences between Reactants and Products (Δ*E*_P–R_, in kcal mol^–1^) for the *S*_0_ Thermolysis Reactions of Clz-Dxt-1 and Clz-Dxt-2 in the Gas Phase at the ωB97XD/6-31+G(d,p) Level of Theory^a^

species	Δ*E*_act_	Δ*E*_P–R_
Clz-Dxt-1	14.3 (12.9)	–100.9 (−100.0)
Clz-Dxt-2	27.2 (25.9)	–89.7 (−88.9)

a Values in parentheses refer to energy re-evaluations at the CAM-B3LYP/6-31+G(d,p) level of theory.

Interestingly, this thermolysis reaction appears to be highly favorable in energetic terms. Namely, it is quite exothermic (by −100.9 kcal mol^–1^) with relatively low Δ*E*_act_, which indicates the energetic feasibility of this thermolysis reaction. This is in line with previous data for the thermochemistry of dioxetanes and dioxetanones.^34^ Interestingly, the low Δ*E*_act_ (14.3 kcal mol^–1^) found for Clz-Dxt-1 is significantly lower than what was found both experimentally and theoretically for different neutral dioxetanes and dioxetanones, which were in the order of ∼20 kcal mol^–1^.^9−12,22,23,25,35,36^ Thus, this reaction appears to be quite feasible, both in absolute and comparative terms. Furthermore, Teranishi et al. did not indicate that the luminescence generated by the thermolysis of Clz-Dxt-1 did not follow a flash-type profile, typical of the chemiluminescence of imidazopyrazinones.^21^ Thus, it is relevant that the energetics here determined are in line with a flash-type profile of chemiluminescence emission.^35,36^

**Figure 2:**
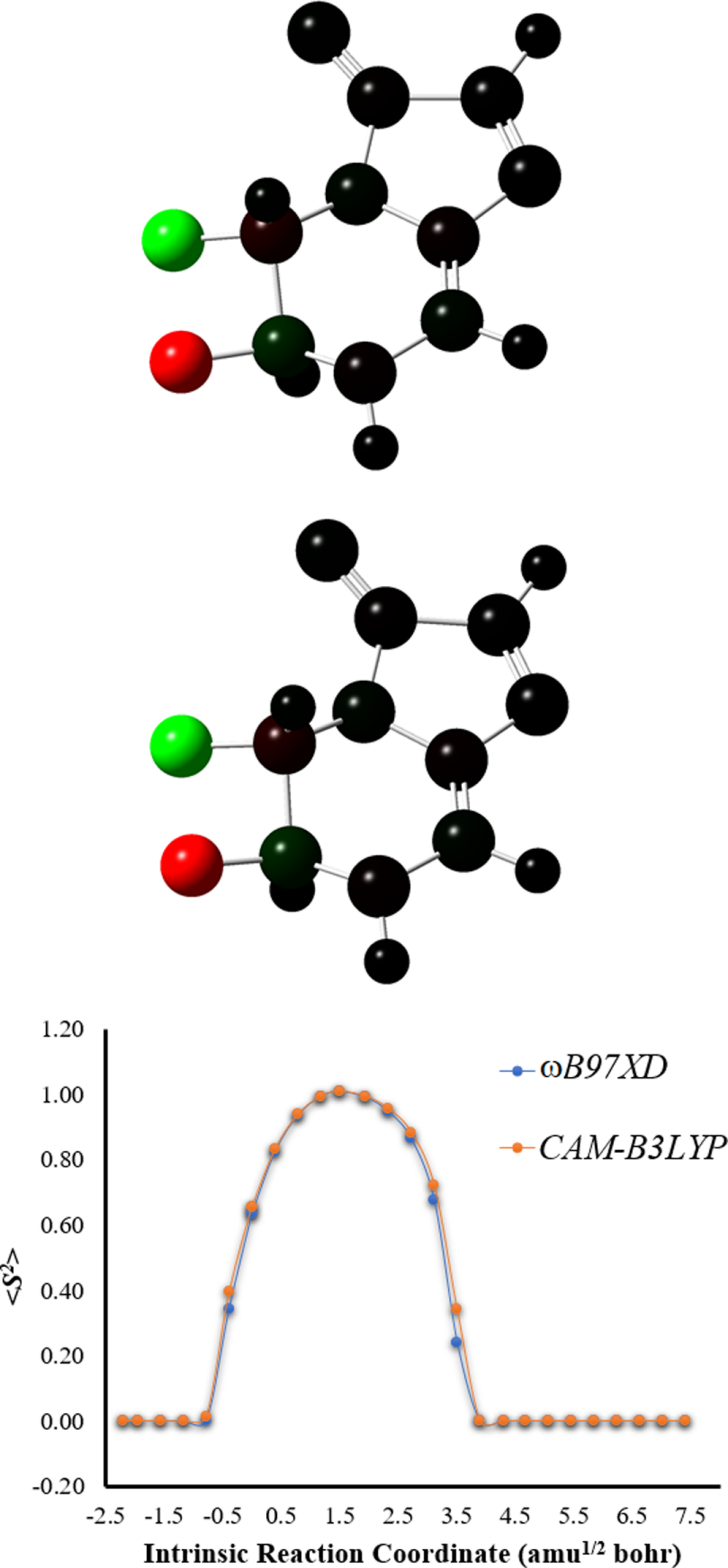
Top and middle: Mulliken spin densities at the *S*_0_ TS structure (intrinsic reaction coordinate of 0.0 amu^1/2^ bohr) of Clz-Dxt-1 calculated with the ωB97XD and CAM-B3LYP functionals (respectively), represented with color atoms by density. Bottom: ⟨*S*^2^⟩ values as a function of intrinsic reaction coordinates of *S*_0_ Clz-Dxt-1 calculated at the ωB97XD/6-31+G(d,p) and CAM-B3LYP/6-31+G(d,p) levels of theory.

Key geometrical parameters for this type of reaction are generally considered to be the bond lengths of the peroxide (O–O) and carbon–carbon (C–C) bonds that constitute the cyclic peroxide ring.^9−13,22−25,34^ Thus, we monitored the changes in the length of these bonds as a function of intrinsic reaction coordinates, which are presented in Figure 1. This reaction follows a typical stepwise mechanism, in which the reaction starts with O–O bond breaking with a minimal C–C bond length variation. C–C bond stretching only starts after the breakage of the O–O bond in the TS region.^9−13,22−25,34^ Evaluation of ⟨*S*^2^⟩ as a function of intrinsic reaction coordinates (Figure 2) indicates that O–O breaking leads to the formation of a biradical species (⟨*S*^2^⟩ values of ∼1), consistent with previous studies on this type of molecules.^9−13,22−25,34^

Analysis of the Mulliken spin density (Figure 2) shows that the spin density is located solely on the two oxygen heteroatoms that constitute the O–O bond, which means that the biradical is formed by homolytic cleavage of that bond without electron transfer (ET) from other moieties of the molecules. Figure 1 also presents the NPA charge separation between the dioxetane and imidazopyrazinone moieties of Clz-Dxt-1 as a function of intrinsic reaction coordinates. There is relevant charge separation between moieties at each reaction coordinate, with dioxetane possessing a positive charge and the imidazopyrazinone moiety possessing a negative charge. However, while there is relevant charge separation between moieties, this separation does not change relevantly as the thermolysis reaction progresses. Thus, our results show that there is no relevant charge transfer (CT) between moieties during the decomposition reaction of Clz-Dxt-1.

The results regarding the lack of ET and CT processes indicate that the unimolecular *S*_0_ thermolysis of Clz-Dxt-1 proceeds without contribution from chemically induced electron-exchange luminescence (CIEEL, ET-based) and charge-transfer-initiated luminescence (CTIL, CT-based) mechanisms.^25,37^

Having characterized the S_0_ thermolysis of Clz-Dxt-1, it is important to ensure that the presented results do not contain functional-dependent errors/artifacts. Thus, we have re-evaluated the *S*_0_ energies with another long-range-corrected functional: CAM-B3LYP.^38^ This functional has also been regularly employed to study dioxetanes and dioxetanones.^11,25,26^ More specifically, single-point calculations were performed at the UCAM-B3LYP/6-31+G(d,p) level of theory on top of *S*_0_ structures obtained at the ωB97XD/6-31G(d,p) level of theory. The associated potential energy curves and variations of ⟨*S*^2^⟩ are shown in Figures S1 and 2, respectively. The Mulliken spin density is presented in Figure 2, while NPA charge separation is presented in Figure S1. Finally, Δ*E*_act_ and Δ*E*_P–R_ are also given in Table 1. The results obtained with both functionals are in high agreement, which supports the validity of our calculations regarding the *S*_0_ thermolysis of Clz-Dxt-1.

After evaluating the *S*_0_ thermolysis, we proceeded to characterize the *S*_0_ → *S*_1_ chemiexcitation for Clz-Dxt-1 at the TD ωB97XD/6-31+G(d,p) level of theory (Figure 1). The obtained potential energy curves are qualitatively similar to what is expected for these systems.^9−13,20,22−25,29−31^ Namely, at the start of the reaction, the *S*_0_–*S*_1_ energy gap is significant but decreases upon entering the biradical region. After the reacting molecules exit that region, the energy gap starts to increase again. However, relevant quantitative differences are observed when we compare these potential energy curves with those obtained at the TD-DFT level of theory for neutral imidazopyrazinone-based dioxetanones. For the latter, the chemiexcitation profile is composed of a large and flat biradical region, where *S*_0_ and *S*_1_ are degenerated/nearly-degenerated (energy gaps lower than ∼10 kcal mol^–1^).^9−12,22,25,29−31^ This type of profile can be associated with efficient chemiexcitation, as there are near-infinite possibilities for nonadiabatic transitions. However, this is not the case with Clz-Dxt-1, as there is no large and flat biradical region of degeneracy (Figure 1). Furthermore, the *S*_0_–*S*_1_ energy gaps are always higher than 12 kcal mol^–1^.

Given this, our results indicate that the *S*_0_ → *S*_1_ chemiexcitation pathway for Clz-Dxt-1 is less efficient than the one found for neutral imidazopyrazinone-based dioxetanones (including that of Coelenterazine)^9−12,22,25,29−31^ To further support our results, we have re-evaluated the *S*_0_ and *S*_1_ energies at the TD CAM-B3LYP/6-31+G(d,p) level of theory (Figure S1). The results are identical, with energy gaps being higher than 11 kcal mol^–1^.

So, our results indicate that one of the reasons for Clz-Dxt-1 being only associated with nonluminescent pathways is due to not possessing an efficient *S*_0_ → *S*_1_ chemiexcitation pathway.^39^ Without a relevant *S*_1_ chemiexcitation yield, there is no formation of enough chemiexcited chemiluminophores to generate appreciable light, which helps to explain the experimental findings associated with this molecule.^21^

Besides *S*_0_ → *S*_1_ chemiexcitation, evaluating the *S*_0_ → *T*_1_ chemiexcitation pathway is also important for this system as it is known that dioxetanes that decompose without relevant ET/CT generate luminophores with a high triplet-to-singlet ratio.^34−36,40^ Furthermore, as phosphorescence is easily quenched in the solution, an efficient *S*_0_ → *T*_1_ chemiexcitation could also explain why the thermolysis of Clz-Dxt-1 is not associated with luminescence.^21^ The potential energy curves for *S*_0_ and *T*_1_ are presented in Figure 1 and were obtained at the ωB97XD/6-31+G(d,p) level of theory. Interestingly, *S*_0_ and *T*_1_ are degenerated during most the biradical region (between coordinates of 0.0 and 2.7 amu^1/2^ bohr), with *S*_0_–*T*_1_ energy gaps of 0.3–4.9 kcal mol^–1^. In fact, the *T*_1_ state became lower in energy than the *S*_0_ state at some reaction coordinates. Thus, intersystem crossing appears to be possible, when considering energy gap-based criteria. Re-evaluations of *S*_0_ and *T*_1_ energies at the CAM-B3LYP/6-31+G(d,p) level of theory (Figure S1) also agree with these results.

Therefore, the different *S*_0_ → *S*_1_ and *S*_0_ → *T*_1_ chemiexcitation profiles (Figures 1 and S1) are in line with experimental findings, which indicate that non-ET/CT-based dioxetanes generate luminophores with higher triplet-to-singlet ratios.^34−36,40^ This feature would also help to explain why Clz-Dxt-1 is associated with low luminescence,^21^ as phosphorescence is easily quenched in a solution.

Having determined the intrinsic pathways and mechanisms for the thermolysis of Clz-Dxt-1, while unperturbed by external effects, it is important to understand if solvent effects can affect the either *S*_0_ → *S*_1_ or *S*_0_ → *T*_1_ chemiexcitation paths. To that end, we re-evaluated the energies of S_0_, *S*_1_, and *T*_1_ states in implicit solvents using the IEFPCM solvent implicit model^41^ at the ωB97XD/6-31+G(d,p) level of theory. Calculations were performed in diethyl ether because its dielectric constant is in line with what is expected for both enzymatic active sites (such as luciferases and photoproteins)^42,43^ and aprotic solvents (as diglyme) where chemiluminescent reactions of Coelenterazine and other imidazopyrazinones can be recorded.^44^ The resulting *S*_0_–*S*_1_ and *S*_0_–*T*_1_ energy gaps, as a function of intrinsic reaction coordinates, are presented in Figure 1.

These energy gaps indicate that the inclusion of solvent effects does not lead to relevant qualitative differences (Figure 1). There is still no large and flat region where *S*_0_ and *S*_1_ states are degenerated, and the associated energy gaps are still quite high. Meanwhile, there are two crossings between *S*_0_ and *T*_1_ states, with the latter becoming even lower in energy than the former at some reaction coordinates (Figure 1).

Nevertheless, there are some relevant quantitative differences. For one, the *T*_1_ is stabilized in implicit diethyl ether, meaning that it becomes even lower than the *S*_0_ state in the solvent than in the gas phase (energy gaps of −13.6 and −3.0 kcal mol^–1^ at some reaction coordinates, respectively). More importantly, in implicit diethyl ether, there is now a reaction coordinate where the *S*_0_–*S*_1_ energy gap is relatively low: 6.9 kcal mol^–1^ at 2.3 amu^1/2^ bohr (Figure 1). Nevertheless, all other points have energy gaps higher than 11 kcal mol^–1^. These data indicates that while *S*_0_ → *S*_1_ chemiexcitation should still be not efficient, in the solvent there is still a point of crossing from *S*_0_ to *S*_1_. This is relevant because while experimental data found Clz-Dxt-1 to decompose with minimal luminescence, some light emission was still detected. So, some minimal pathways for chemiexcitation were needed to be found.

**Figure 3:**
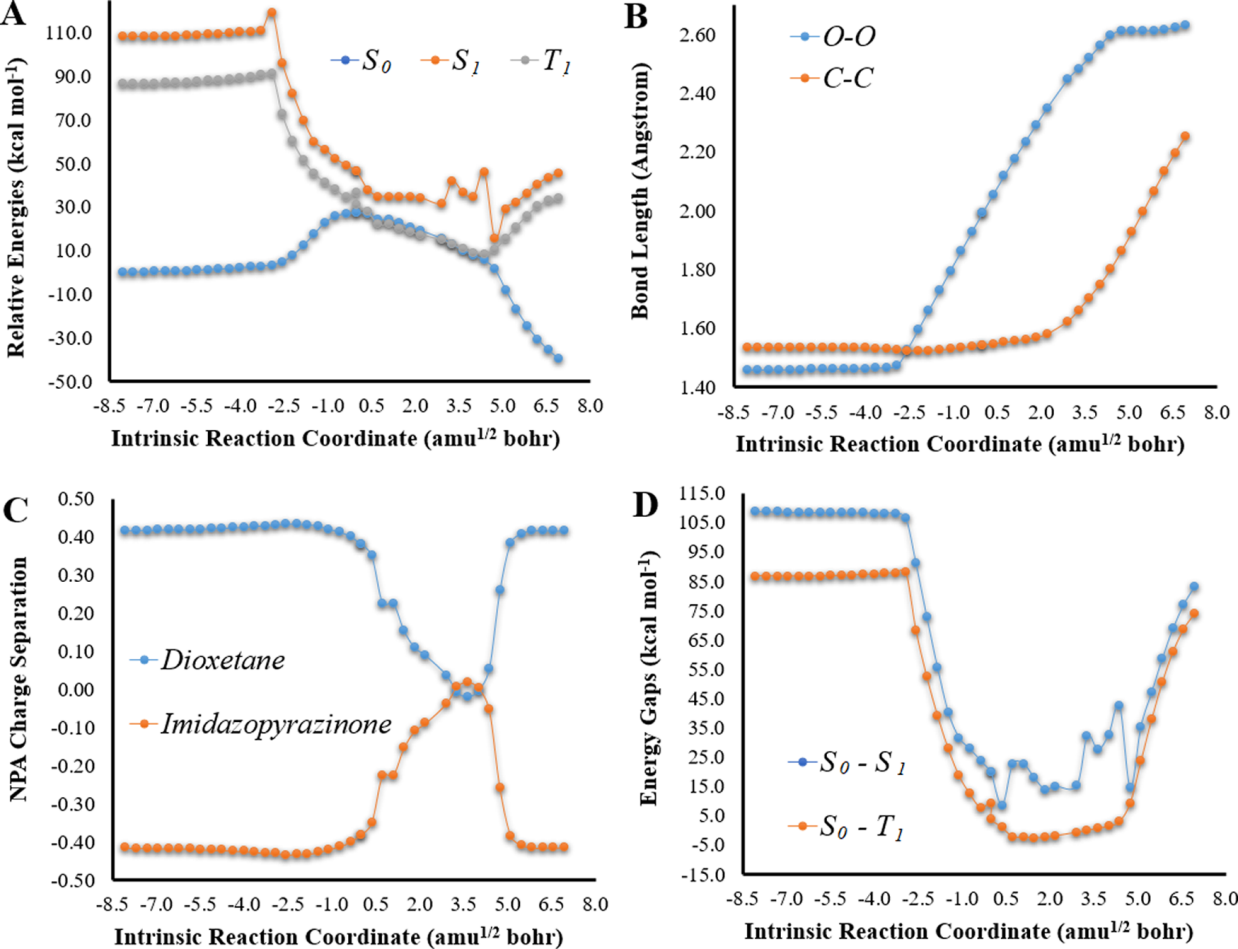
(A) Potential energy curves for the *S*_0_, *S*_1_, and *T*_1_ states of Clz-Dxt-2 in the gas phase, as a function of intrinsic reaction coordinates, at the (TD) ωB97XD/6-31+G(d,p) level of theory. (B) Bond length variations for O–O and C–C bonds of the dioxetane ring for Clz-Dxt-2, as a function of intrinsic reaction coordinates, at the ωB97XD/6-31G(d,p) level of theory. (C) NPA *S*_0_ charge separation between the dioxetane and imidazopyrazinone moieties of Clz-Dxt-2, as a function of intrinsic reaction coordinates, at the ωB97XD/6-31+G(d,p) level of theory. (D) *S*_0_–*S*_1_ and *S*_0_–*T*_1_ energy gaps (in kcal mol^–1^) for Clz-Dxt-2 in implicit diethyl ether, as a function of intrinsic reaction coordinates, at the (TD) ωB97XD/6-31+G(d,p) level of theory.

**Figure 4:**
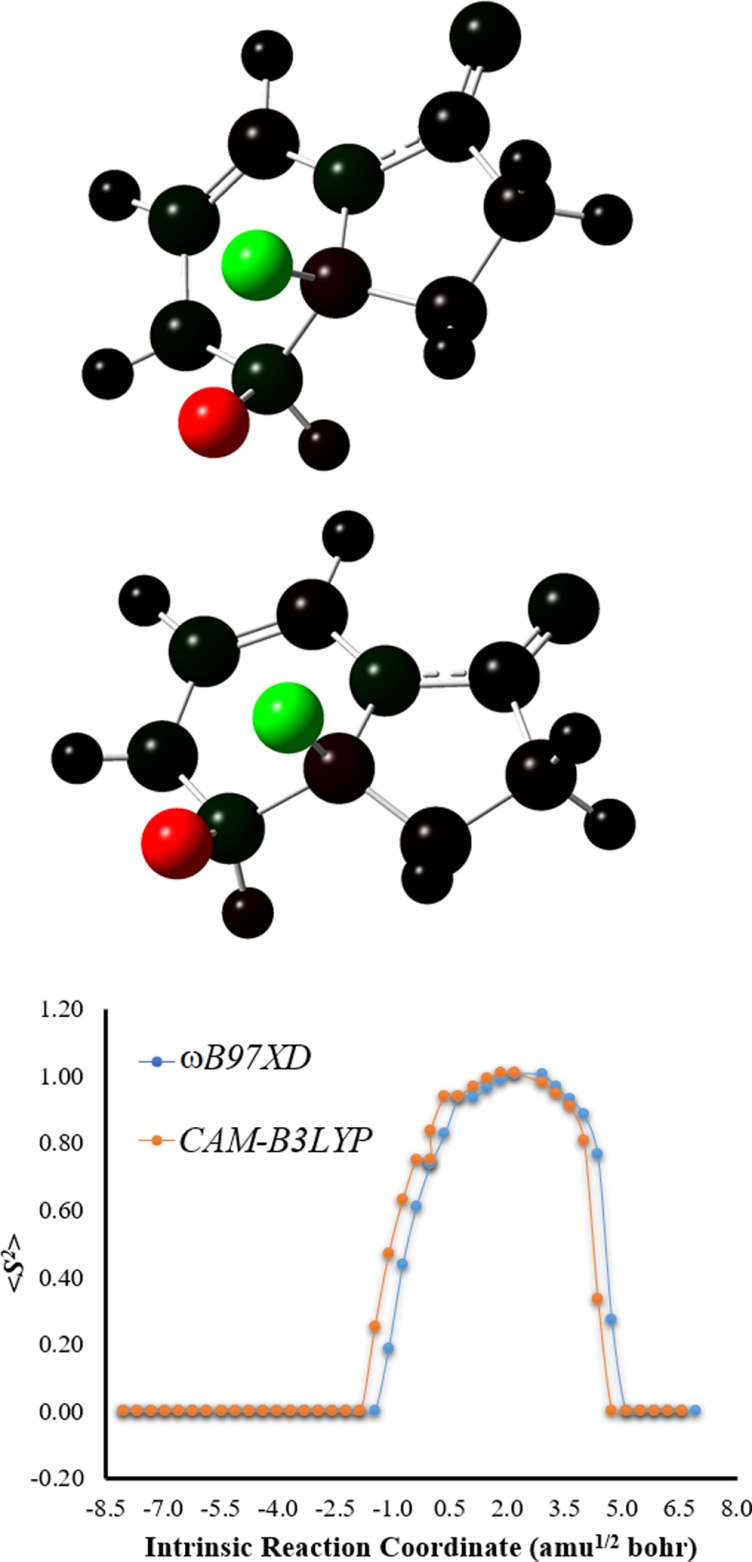
Top and middle: Mulliken spin densities at the *S*_0_ TS structure (intrinsic reaction coordinate of 0.0 amu^1/2^ bohr) of Clz-Dxt-2 calculated with the ωB97XD and CAM-B3LYP functionals (respectively), represented with colored atoms by density. Bottom: ⟨*S*^2^⟩ values as a function of intrinsic reaction coordinates of *S*_0_ Clz-Dxt-2 calculated at the ωB97XD/6-31+G(d,p) and CAM-B3LYP/6-31+G(d,p) levels of theory.

After investigating Clz-Dxt-1, we shifted our focus to the characterization of Clz-Dxt-2 (Scheme 1). The potential energy curves for *S*_0_, *S*_1_, and *T*_1_ states, as a function of intrinsic reaction coordinates, are shown in Figure 3. They were obtained at the (TD) ωB97XD/6-31+G(d,p) level of theory. The variation of important geometrical parameters (O–O and C–C bonds) and ⟨*S*^2^⟩ is also shown in Figures 3 and 4 (respectively), while associated Δ*E*_act_ and Δ*E*_P–R_ in Table 1.

There are relevant energetic differences between Clz-Dxt-1 and Clz-Dxt-2 (Table 1). More specifically, the thermolysis reaction of the latter presents a higher Δ*E*_act_ (27.2 kcal mol^–1^) than the former (14.3 kcal mol^–1^) while being relevantly less exothermic (by −89.7 kcal mol^–1^). Re-evaluation of *S*_0_ energies at the CAM-B3LYP/6-31+G(d,p) level of theory revealed a slightly lower Δ*E*_act_ (25.9 kcal mol^–1^) for Clz-Dxt-2 (Table 1). While the Δ*E*_act_ values for Clz-Dxt-2 appear high, it should be noted that Nery et al. already found activation energies of ∼25 kcal mol^–1^ for neutral sililoxyphenyl-substituted dioxetanes,^26,35^ while Adam and Baader measured activation energies between ∼23 and ∼28 kcal mol^–1^ for simpler methylated dioxetanes.^40^ So, these values are in line with experimental data. Furthermore, the thermolysis of Clz-Dxt-2, as proposed by Burakova et al.,^17^ proceeds inside a photoprotein under incandescent light irradiation, which could catalyze the reaction. Finally, limited data was provided by Burakova et al. regarding the kinetics and yields associated with the thermolysis of Clz-Dxt-2. Thus, given that the reaction is still quite exothermic, the higher Δ*E*_act_ values should not prevent the feasibility of this reaction. It is also interesting to note that while Clz-Dxt-1 and Clz-Dxt-2 are like mirror structures, their *S*_0_ energetics are quite different.

To elucidate the thermolysis mechanism of Clz-Dxt-2, we monitored the associated changes of length of the O–O and C–C bonds of the peroxide ring as a function of intrinsic reaction coordinates (Figure 3). The thermolysis mechanism was found to be the same as for Clz-Dxt-1 and other cyclic peroxides.^9−13,22−25,34^ Namely, it follows a stepwise pathway that starts with O–O bond breaking, followed by C–C bond rupture. Evaluation of ⟨*S*^2^⟩ as a function of intrinsic reaction coordinates also revealed the presence of a biradical during peroxide ring rupture (Figure 4).^9−13,22−25,34^ Evaluation of the Mulliken spin density (Figure 4) revealed that the formation of the biradical results from homolytic cleavage of the peroxide bond, in agreement with the decomposition of Clz-Dxt-1.

The NPA charge separation between the dioxetane and imidazopyrazinone moieties of Clz-Dxt-2, as a function of intrinsic reaction coordinates, is presented in Figure 3. Interestingly and contrary to Clz-Dxt-1, there is now relevant CT and back CT (BCT) during the decomposition of Clz-Dxt-2. More specifically, while the dioxetane is generally positively charged and the imidazopyrazinone group is negatively charged, there is transfer of negative charge from the latter to the former between intrinsic coordinates of 0.4 and 4.0 amu^1/2^ bohr. This leads to the two fragments becoming neutral, upon occurrence of BCT that re-establish the charge separation between moieties. Thus, our results indicate that contrary to the case of Clz-Dxt-1, the *S*_0_ thermolysis of Clz-Dxt-1 proceeds with contribution of the CTIL (CT-based) mechanism.^25,37^

It should be noted that the CTIL mechanism is typically associated with low Δ*E*_act_, while non-CTIL-based thermolysis is associated with higher Δ*E*_act_.^10,11,22,25,35,36,45^ However, this is the exact opposite to what was observed here, as the Δ*E*_act_ of Clz-Dxt-2 is significantly higher than that of Clz-Dxt-1. This can be explained by the fact that in typical CTIL mechanisms, the initial CT step is coupled with peroxide bond breaking, being already observed in the TS structure, thereby helping to decrease Δ*E*_act_.^10,11,22,25,35,36,45^ In the case of Clz-Dxt-2, the initial CT step is not coupled with peroxide bond breaking, as CT only occurs after reaching the TS. Thus, CT provides no benefit to the Δ*E*_act_ of Clz-Dxt-2.

As in the case of Clz-Dxt-1, we re-evaluated the *S*_0_ energies of Clz-Dxt-2 with CAM-B3LYP. The associated potential energy curves and variations of ⟨*S*^2^⟩ are shown in Figures S2 and 4, respectively. The NPA charge separation is presented in Figure S2. Δ*E*_act_ and Δ*E*_P–R_ are also given in Table 1. Once again, there are no relevant differences between the results obtained with CAM-B3LYP and ωB97XD, supporting the validity of the present calculations.

The next step in the study of Clz-Dxt-2 was the characterization of the *S*_0_ → *S*_1_ chemiexcitation at the TD ωB97XD/6-31+G(d,p) level of theory (Figure 3). Similar to Clz-Dxt-1, the singlet chemiexcitation profile of Clz-Dxt-2 does not appear to be particularly efficient. Contrary to neutral imidazopyrazinone-based dioxetanones,^9−12,22,25,29−31,45^ there is not a large and flat biradical region where *S*_0_ and *S*_1_ are degenerated. In fact, the *S*_0_–*S*_1_ energy gaps for Clz-Dxt-2 are always higher than 10.5 kcal mol^–1^, with energy gaps for neutral imidazopyrazinone-based dioxetanones being significantly lower than ∼10 kcal mol^–1^ during large portions of their potential energy surfaces.^9−12,22,25,29−31,45^ Re-evaluation of the *S*_0_ and *S*_1_ energies at the TD CAM-B3LYP/6-31+G(d,p) level of theory (Figure S2) provided identical results, with energy gaps being higher than 11.1 kcal mol^–1^. Thus, these results agree with the experimental work of Burakova et al.,^17^ in which the thermolysis of Clz-Dxt-2 was associated with the “dark” formation of degradation products, as the *S*_0_ → *S*_1_ chemiexcitation of this dioxetane should not be very efficient.

Interestingly, the thermolysis of Clz-Dxt-2 proceeded with CTIL character, with the CTIL mechanism being used to explain efficient chemiexcitation.^11,25,26^ However, and despite the observation of CT/BCT, the chemiexcitation profile of Clz-Dxt-2 appears to be inefficient. Even more, the chemiexcitation profiles of Clz-Dxt-1 and Clz-Dxt-2 are quite similar, despite the thermolysis of the former proceeding without CTIL character, contrary to the case of the latter. Thus, these results are in line with the previous work of our group, in which no clear relationship between CTIL and the chemiexcitation of high-energy peroxides was found.^23,45^

We also calculated the potential energy curves for the *S*_0_ → *T*_1_ chemiexcitation of Clz-Dxt-2 at the ωB97XD/6-31+G(d,p) level of theory (Figure 3). Interestingly, *S*_0_ and *T*_1_ are degenerated during most the biradical region (between coordinates of 0.0 and 4.4 amu^1/2^ bohr), with *S*_0_–*T*_1_ energy gaps as low as 0.0–3.9 kcal mol^–1^. In fact, the *T*_1_ state became lower in energy than the *S*_0_ state at some reaction coordinates. Thus, intersystem crossing appears to be possible, when considering energy gap-based criteria. Re-evaluations of *S*_0_ and *T*_1_ energies at the CAM-B3LYP/6-31+G(d,p) level of theory (Figure S2) also agree with these results. Thus, the triplet chemiexcitation profile of Clz-Dxt-2 is identical to that of Clz-Dxt-1, in energetic terms, providing another nonluminescent pathway for this thermolysis reaction.

Finally, we re-evaluated the energies of S_0_, *S*_1_, and *T*_1_ states for Clz-Dxt-2 in implicit diethyl ether at the ωB97XD/6-31+G(d,p) level of theory (Figure 3). As for Clz-Dxt-1, the inclusion of solvent effects did not induce relevant qualitative differences between the chemiexcitation profiles of Clz-Dxt-2 in gas and condensed phases (Figure 3). Nevertheless, some relevant quantitative differences can be found for singlet chemiexcitation, as the inclusion of solvent effects leads to a relevantly lower *S*_0_–*S*_1_ energy gap at one specific reaction coordinate: 8.2 kcal mol^–1^ at 0.4 amu^1/2^ bohr. This energy gap could allow for some chemiexcitation, but with minimal luminescence as the energy gaps for other coordinates are always higher than ∼14 kcal mol^–1^. As for triplet chemiexcitation, inclusion of solvent effects induced neither relevant quantitative nor qualitative differences in the *S*_0_–*T*_1_ energy gaps. However, that was not the case for Clz-Dxt-1, in which the *T*_1_ was significantly stabilized in diethyl ether. Thus, while the *T*_1_ state of Clz-Dxt-1 appears to be sensitive to the microenvironment, the *T*_1_ state of Clz-Dxt-2 does not. This means that there are relevant differences in thermolysis and chemiexcitation properties of Coelenterazine dioxetanes.

The final step in this study was to try to assess the intersystem crossing efficiencies associated with *T*_1_ chemiexcitation for each dioxetane. This was performed qualitatively by considering the El-Sayed rule, in which the rate of intersystem crossing is relatively large when the transition is associated with a change of orbital type.^46^ Orbitals were obtained by DFT calculations with Gaussian 09 but are visualized through Avogadro software.^47^

For Clz-Dxt-1, we analyzed the intersystem crossing at the reaction coordinates where the *S*_0_–*T*_1_ energy gap initially reaches closer to 0 kcal mol^–1^(indicating reaching to the region of intersystem crossing, Figure 1): 0.7 kcal mol^–1^ at 0.4 amu^1/2^ bohr, The analysis was performed by comparing the HOMO and LUMO orbitals of the *S*_0_ state with the SOMO(1) and SOMO(2) orbitals of the *T*_1_ state, respectively.^29^ There indeed was observed variations in the orbitals involved with the *S*_0_ and *T*_1_ states (Figure S3) of Clz-Dxt-1. Namely, the HOMO and LUMO orbitals of the *S*_0_ states appear to be π and σ* ones, respectively. Meanwhile, the SOMO(1) and SOMO(2) of the *T*_1_ state appear to be *n* and π ones. Therefore, these results indicate some associated efficiency with intersystem crossing at this point.

The same analysis was performed for Clz-Dxt-2 at the reaction coordinate of 0.4 amu^1/2^ bohr (*S*_0_–*T*_1_ energy gap of 1.0 kcal mol^–1^). The HOMO and LUMO orbitals of the *S*_0_ state of Clz-Dxt-2 (Figure S4) are similar to those found for Clz-Dxt-1 (Figure S3), being also π and σ* orbitals (respectively). Meanwhile, the SOMO(1) and SOMO(2) orbitals of the *T*_1_ state of Clz-Dxt-2 appear to be *n* and π ones (the same type as the ones found for Clz-Dxt-1, in Figure S3). Thus, it appears that the intersystem crossing for Clz-Dxt-2 should be associated with some efficiency.

The indication that the initial point of intersystem crossing is associated with some efficiency also helps to explain why these molecules are related with inefficient luminescence, as it should lead to higher triplet-to-singlet product ratios.

## Conclusions

Here, we investigated for the first time the thermolysis mechanisms and chemiexcitation profiles of two Coelenterazine-based dioxetanes by employing a theoretical approach based on TD-DFT calculations. Similar to Coelenterazine and other imidazopyrazinone-based dioxetanones, the thermolysis of these dioxetanes proceeds via similar stepwise biradical mechanisms, which are significantly exothermic. However, the activation energies of the two Coelenterazine-based dioxetanes differ significantly between themselves by about ∼13 kcal mol^–1^. In fact, while Clz-Dxt-2 presents activation parameters similar to neutral imidazopyrazinone-based dioxetanones, the energetics of Clz-Dxt-1 are closer to anionic dioxetanones. Analysis of the singlet chemiexcitation profile of these dioxetanes revealed that, and contrary to imidazopyrazinone-based dioxetanones, there is no region of degeneracy between *S*_0_ and *S*_1_ states where chemiexcitation can occur efficiently. Thus, these Coelenterazine dioxetanes can only be associated with minimal singlet chemiexcitation. On the contrary, the *S*_0_ and *T*_1_ states of these molecules are degenerated within the biradical region of the thermolysis reaction, providing a way for nonluminescent triplet chemiexcitation. Thus, and in agreement with experimental findings, our results indicate that these dioxetanes can provide “dark” pathways for the formation of nonluminescent degradation products during the chemi- and bioluminescent reactions of Coelenterazine and other imidazopyrazinones, which can be found in eight phyla of bioluminescent organisms.

## Supporting Information Available

Cartesian coordinates of TS structures for Clz-Dxt-1 and Clz-Dxt-2; *S*_0_, *S*_1_, and *T*_1_ energies as a function of intrinsic reaction coordinates at the (TD) CAM-B3LYP level of theory; NPA charge separation as a function of intrinsic reaction coordinates at the CAM-B3LYP level of theory; and HOMO/LUMO and SOMO(1)/SOMO(2) orbitals for Clz-Dxt-1 and Clz-Dxt-2 at the initial point for intersystem crossing (PDF)
