# Supplementary material for: Theoretical Study of the Thermolysis Reaction and Chemiexcitation of Coelenterazine Dioxetanes
Source: J Phys Chem A. 2022 May 25;126(22):3486–94. doi: 10.1021/acs.jpca.2c01835 (PMC9776548; doi:10.1021/acs.jpca.2c01835)
Supplement: Supplementary file 1 — jp2c01835_si_001.pdf [file jp2c01835_si_001.pdf]

THEORETICAL STUDY OF THE THERMOLYSIS REACTION AND CHEMIEXCITATION OF COELENTERAZINE DIOXETANES

Carla M. Magalhães[1], Joaquim C.G. Esteves da Silva[1,2] and Luís Pinto da Silva[1,2,*]

[1] Chemistry Research Unit (CIQUP), Institute of Molecular Sciences (IMS), Faculty of Sciences of University of Porto (FCUP), Rua do Campo Alegre 687, 4169-007 Porto, Portugal.

[2] LACOMEPHI, GreenUPorto, Department of Geosciences, Environment and Territorial Planning, Faculty of Sciences of University of Porto (FCUP), Rua do Campo Alegre 687, 4169-007 Porto, Portugal.

Table S1. Cartesian coordinates of the TS structure for the thermolysis of Clz-Dxt-1, obtained in the gas phase at the ωB97XD/6-31G(d,p) level of theory.

O	-0.40761	2.88088	0.07808
C	0.36728	1.96348	-0.06901
C	1.85930	1.95283	-0.21690
N	2.36670	0.76650	-0.19342
C	1.29728	-0.11518	-0.08992
N	0.07497	0.60454	-0.13052
C	1.38281	-1.44188	0.16098
N	0.25457	-2.23985	0.37491
C	-0.89673	-1.55122	-0.15146
C	-0.98666	-0.16713	0.44426
O	-2.33735	-0.03502	0.07030
O	-2.20946	-1.88426	0.23134
H	2.46060	2.85045	-0.29733
H	2.35610	-1.90865	0.23025
H	0.36430	-3.20576	0.09470
H	-0.84390	-1.46651	-1.24724
H	-0.90429	-0.19331	1.53797

Table S2. Cartesian coordinates of the TS structure for the thermolysis of Clz-Dxt-2, obtained in the gas phase at the ωB97XD/6-31G(d,p) level of theory.

C	2.05587	-1.21446	-0.30517
C	1.96436	0.28292	-0.05438
N	0.61895	0.55315	0.10530
C	-0.18707	-0.61839	0.03886
N	0.67116	-1.60104	-0.56502
O	2.85658	1.09549	-0.00339
C	0.02836	1.81431	0.23059
C	-1.28049	1.93659	-0.02685
N	-2.07433	0.90486	-0.50663
C	-1.57261	-0.44638	-0.61560
O	-0.66227	-0.94309	1.32571
O	-2.25776	-1.34471	0.20284
H	2.50128	-1.69535	0.57550
H	2.70368	-1.40239	-1.16467
H	0.43077	-2.52187	-0.22054
H	0.67426	2.62282	0.53639
H	-1.78046	2.88810	0.10029
H	-3.04554	0.93725	-0.23201
H	-1.58791	-0.80998	-1.65105

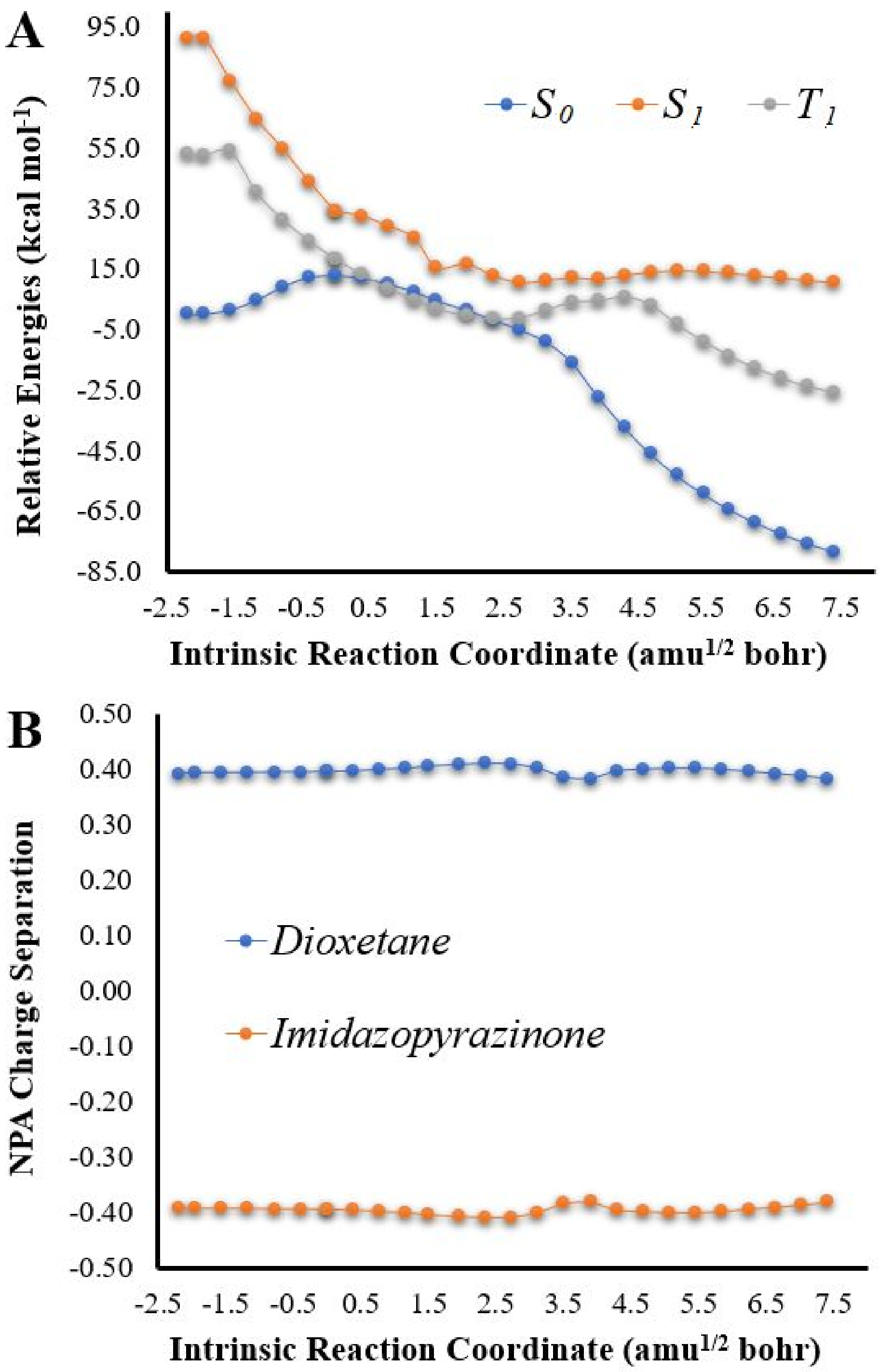

Figure S1. A - Potential energy curves for the S_0, S_1 and T_1 states of Clz-Dxt-1 in the gas phase, as a function of intrinsic reaction coordinates, at the (TD) CAM-B3LYP/6-31+G(d,p) level of theory. B – NPA S_0 charge separation between the dioxetane and imidazopyrazinone moieties of Clz-Dxt-1, as a function of intrinsic reaction coordinates, at the CAM-B3LYP/6-31+G(d,p) level of theory.

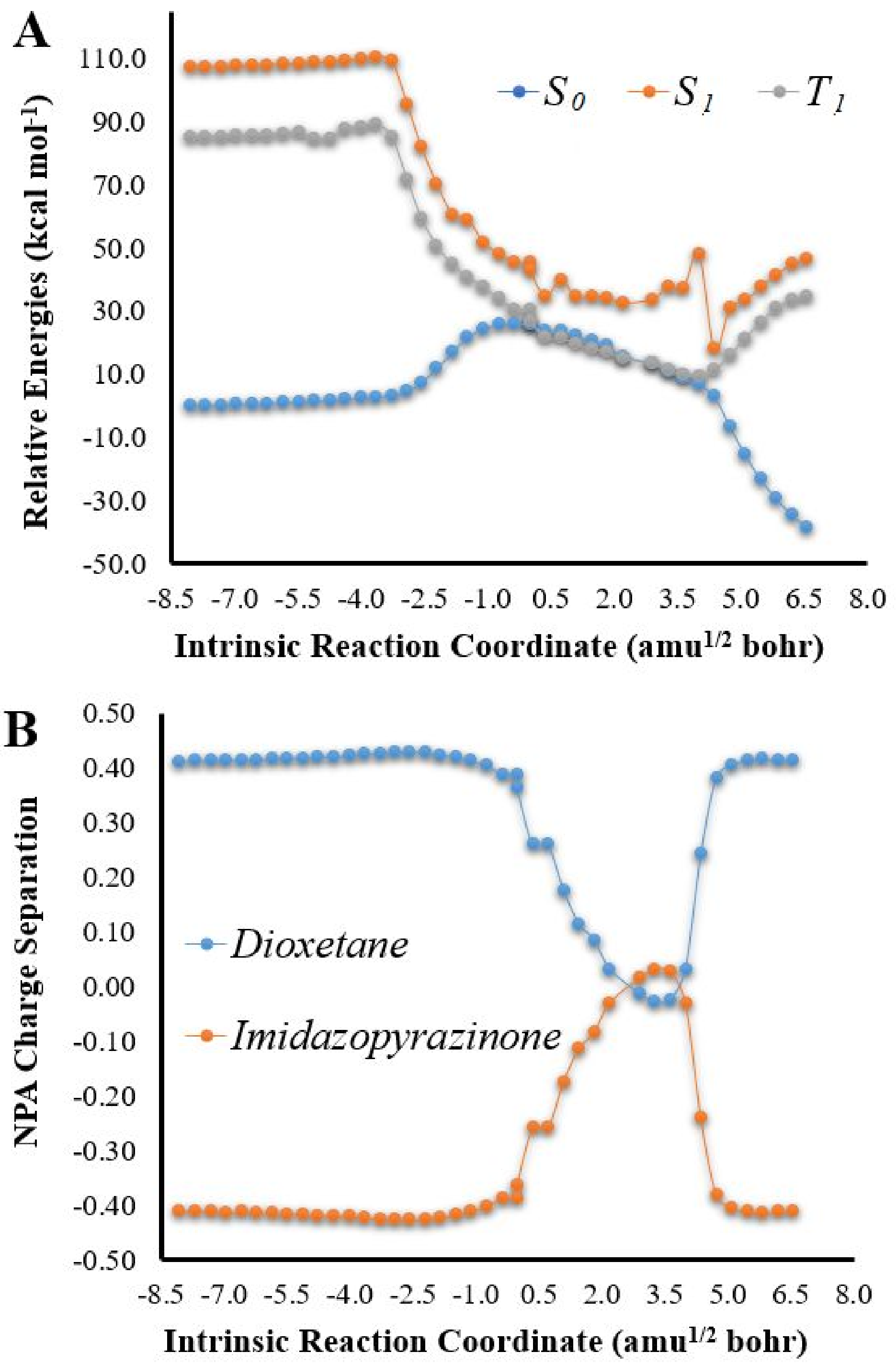

Figure S2. A - Potential energy curves for the S_0, S_1 and T_1 states of Clz-Dxt-2 in the gas phase, as a function of intrinsic reaction coordinates, at the (TD) CAM-B3LYP/6-31+G(d,p) level of theory. B – NPA S_0 charge separation between the dioxetane and imidazopyrazinone moieties of Clz-Dxt-2, as a function of intrinsic reaction coordinates, at the CAM-B3LYP/6-31+G(d,p) level of theory.

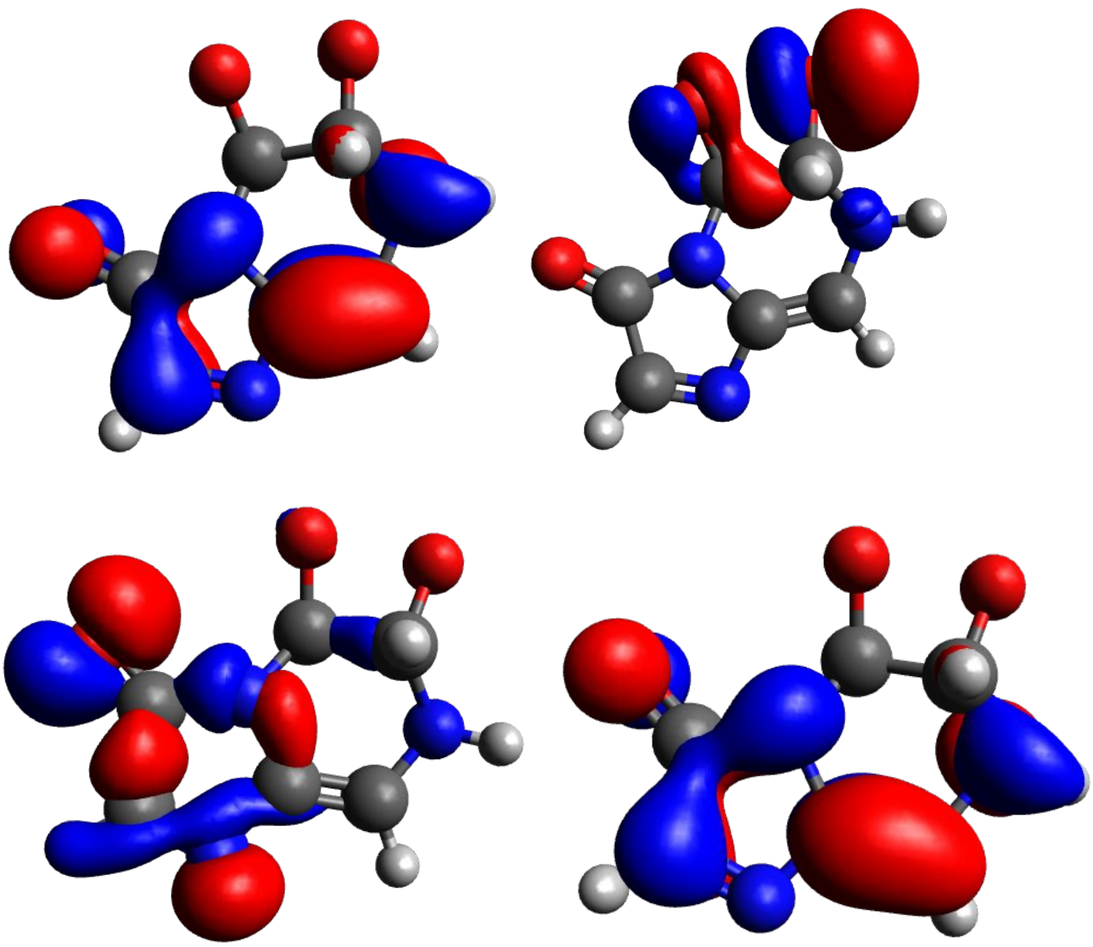

Figure S3. HOMO (top-left) and LUMO (top-right) orbitals of the S_0 state of Clz-Dxt-1 *in vacuo*, calculated at the ωB97XD/6-31+G(d,p) level of theory. SOMO1 (bottom-left) and SOMO2 (bottom-right) orbitals of the T_1 state of Clz-Dxt-1 *in vacuo*, calculated at the ωB97XD/6-31+G(d,p) level of theory. At reaction coordinate of 0.4 amu$^{1/2}$ bohr.

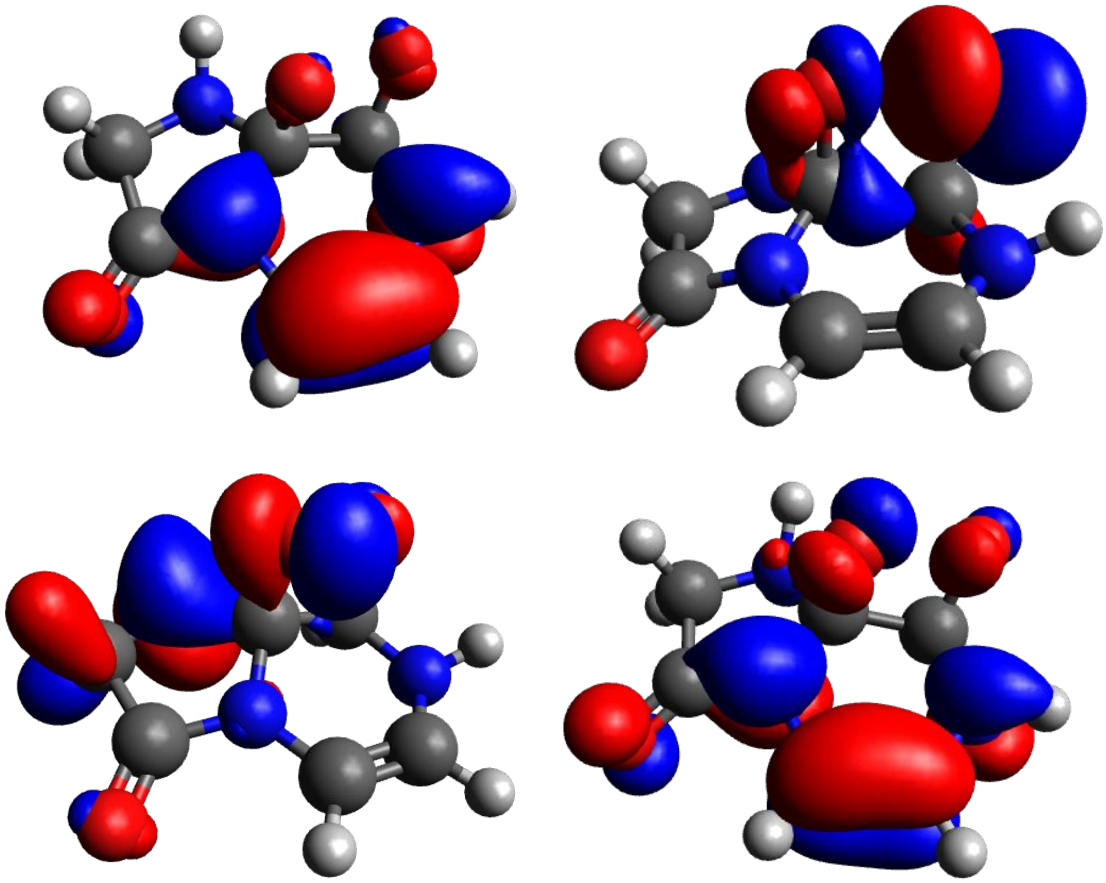

Figure S4. HOMO (top-left) and LUMO (top-right) orbitals of the S_0 state of Clz-Dxt-2 *in vacuo*, calculated at the ωB97XD/6-31+G(d,p) level of theory. SOMO1 (bottom-left) and SOMO2 (bottom-right) orbitals of the T_1 state of Clz-Dxt-2 *in vacuo*, calculated at the ωB97XD/6-31+G(d,p) level of theory. At reaction coordinate of 0.4 amu$^{1/2}$ bohr.
